# Facilitators and barriers in using barcode technology to ensure safe medication dispensing, preparation, and administration in a children's hospital: a focus group study for clinical pharmacists

**DOI:** 10.1007/s11096-026-02132-0

**Published:** 2026-04-02

**Authors:** Laura Laakkonen, Kirsi Kvarnström, Katja Janhunen, Carita Linden-Lahti, Sini Kuitunen

**Affiliations:** 1 Division of Pharmacology and Pharmacotherapy, Faculty of Pharmacy, University of Helsinki, Helsinki, Finland; 2 HUS Pharmacy, Helsinki University Hospital (HUS), Helsinki, Finland; 3 Corporate Group Administration, Western Uusimaa Wellbeing Services County, Finland

**Keywords:** Barcode medication administration, Barcode technology, Focus group study, Medication safety, Pediatrics, Qualitative content analysis

## Abstract

**Introduction:**

Barcode technology is widely used in hospitals to improve medication safety. Although this technology is considered effective for making system-wide improvements, its implementation faces several challenges. Hospital pharmacists play a key role in supporting this process.

**Aim:**

To explore clinical pharmacists’ perceptions of the facilitators and barriers to using barcode technology in a pediatric hospital setting.

**Method:**

A qualitative focus group study. Fourteen clinical pharmacists working in the pediatric department of a university hospital were chosen for the focus groups (n = 3) using purposive sampling to identify the individuals who regularly use barcode technology in clinical practice. The focus group discussions, guided by a semi-structured interview guide with six questions, were recorded and transcribed verbatim. Two researchers independently conducted inductive content analysis, which was later thoroughly reviewed by the entire research group.

**Results:**

The data revealed four main themes: factors encouraging the adoption of barcode technology, factors complicating barcode workflow, ideas to improve workflow efficiency, and at-risk behaviors. Factors encouraging the adoption of barcode technology were associated with the benefits and usability of barcode technology, increased expertise and teamwork, positive user experience, and supportive functions of the electronic health record (EHR) system. Factors complicating barcode workflow included deficiencies related to barcodes in labels and drug packages, negative attitudes of users, organizational factors, the use of the EHR system, and deficiencies in workstations and equipment. These factors were found to contribute to at-risk behaviors, while ideas to improve workflow efficiency focused on removing factors complicating barcode workflow and reducing at-risk behaviors.

**Conclusion:**

Various factors can influence the implementation of barcode technology in clinical practice, underscoring the importance of an organizational process to identify system deficiencies and continuously improve usability. Building on previous studies, our research emphasized the issues related to dispensing and preparation workflows, as well as the need for pediatric-specific EHR system customization as key development areas. Our findings can guide risk management efforts in implementing and maintaining barcode technology in hospitals.

**Supplementary Information:**

The online version contains supplementary material available at 10.1007/s11096-026-02132-0.

## Impact statements

- Key factors encouraging the adoption of barcode technology include aspects of safety, reliability, and user experience.
- Factors complicating barcode workflow expose users to workarounds that involve both system-based and user-oriented at-risk behaviors.
- User engagement in barcode technology development is a key area for future work, as focus groups generated several ideas to address barriers and improve workflow efficiency.
- While barcode technology is widely recommended as a systemic defense with notable advantages, effective implementation of these workflows requires organizational commitment to ongoing risk management.
- Our findings highlight the role of clinical pharmacists in problem-solving, workflow design, and establishing a foundation for barcode workflows by ensuring order accuracy.

## Introduction

Children are a vulnerable patient group with diverse and unique characteristics, making medication management complex and increasing the risk of medication errors (MEs) [1–3]. However, many MEs can be prevented by implementing systemic defenses as part of organizational medication risk management [1, 3, 4]. The most effective error-reduction strategies focus on modifying the systems in which individuals operate, aiming to eliminate the risk of MEs and associated harm [5]. In hospitals, barcode technology is widely recommended to ensure the safe dispensing, preparation, and administration of medications [6–12]. It has been shown to prevent various types of MEs, including incorrect doses, wrong drugs, errors involving the wrong patient, unauthorized medications, and incorrect administration routes. [6, 13–15]. Although barcode technology is generally effective at enhancing medication safety, its implementation is associated with several challenges related to technology and human factors [6, 15–17].

Barcode technology is recommended for pediatric hospitals, but research on its effectiveness in this specialized setting remains limited [18–20]. The pediatric medication management process poses specific challenges that complicate the usability and integration of barcode technology into standard clinical practice [20, 21]. These include issues related to individualized dosing, barcode contamination from liquid oral medications, and the need for smaller patient armbands for the youngest children. Furthermore, challenges associated with adult care settings—such as limited availability and usability of hardware (e.g., mobile computer stations, printers), barcode issues (e.g., unreadable or missing barcodes on drug packages), and the need to replace patient wristbands during care transitions—are also present in pediatrics [17, 21–23]. However, a more comprehensive explanation of the specific considerations for using barcode technology in pediatric hospitals is still needed.

Incorporating barcode technology into routine clinical practice requires adjustments to workflows, ongoing maintenance of the electronic health record (EHR) system, risk management protocols, and staff training [17]. Consequently, research is crucial to identify facilitators and barriers to the effective use of barcode technology, guiding hospitals in developing strategies to realize its benefits. Still, only a few qualitative studies have examined the facilitators and barriers affecting the use of this technology [24–26], and even fewer focus on pediatrics. Clinical pharmacists’ comprehensive expertise in safe medication management processes offers significant potential for establishing and improving barcode workflows [20]; however, only a limited number of studies have explored their perspectives [27, 28].

### Aim

This study aimed to examine clinical pharmacists' perceptions of facilitators and barriers to using barcode technology in a pediatric hospital.

## Method

### Study design

This research was conducted as a qualitative focus group study in a pediatric hospital. The focus group design was chosen because it has proven to be a valuable method for exploring individuals' beliefs, behaviors, and attitudes toward a previously little-studied phenomenon [29, 30]. Qualitative research methods, such as focus groups, can be used to understand specific human phenomena, including the implementation decisions related to new technologies and workflows [30, 31]. The Consolidated Criteria for Reporting Qualitative Research (COREQ) checklist was used in the study reporting (Supplementary file 1) [29].

### Study setting

**Fig. 1:**
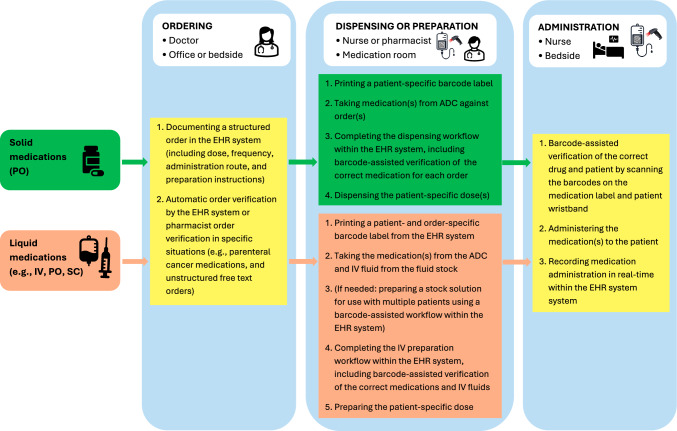
The study site used barcode-assisted workflows for medication dispensing, preparation, and administration. ADC = automated dispensing cabinet, EHR = electronic health record system, IV = intravenous, PO = oral, SC = subcutaneous

Our study was conducted at the Department of Children and Adolescents at HUS Helsinki University Hospital (HUS) in Finland. This department provides specialized healthcare services for all pediatric age groups and disease categories up to age 16. At the time of data collection in 2023, the department had approximately 200 beds across two hospital sites and employed 19 pediatric clinical pharmacists. Pediatric clinical pharmacists were responsible for developing medication safety protocols, managing care unit-based medication inventories, preparing patient-specific parenteral medication doses, conducting medication reconciliation, providing patient counseling, and providing drug information. At the study site, nurses and pharmacists primarily dispensed and prepared patient-specific medications in the care units. In 2020, a new EHR system (Apotti), based on Epic Systems, was introduced in pediatrics and included barcode technology to enhance the safe dispensing, preparation, and administration of medications (Fig. 1). Barcode-assisted workflows were used in all pediatric wards (n = 6), outpatient infusion clinics (n = 2), and intensive care or step-down units (n = 3).

### Study participants

We used purposive sampling to select clinical pharmacists for the study. Participation was voluntary, and pharmacists were recruited via email or in person during pediatric clinical pharmacy team meetings. A prerequisite for participation was that participants either personally used barcode workflows or were otherwise involved in their daily application in the care units. Four pharmaceutical employees did not use barcode technology, making them ineligible for inclusion in the study sample. Except for one participant who withdrew from the study due to changes in their work situation, all clinical pharmacists who met the inclusion criteria participated in the focus group discussions.

### Data collection and preprocessing

The study material was collected in three focus group discussions in January 2023. The principal investigator (LL, pharmacist, BSc) facilitated the interview sessions, with assistance from another investigator (SK, pharmacist, PhD). Both investigators had received training in facilitating focus group discussions and had experience in hospital pharmacy as a trainee (LL) or as a senior clinical pharmacist in pediatrics (SK). The principal investigator had no personal connection with the participants; however, the assistant researcher worked with them. The participants received a written statement about the study and provided written informed consent to participate before the focus group discussions. Participants were informed at the beginning of the interviews that the research material would be handled anonymously and confidentially. The research group (LL, KK, KJ, CL-L, SK) had experience planning and facilitating focus group discussions, conducting qualitative analysis, using barcode technology, and working with pediatrics.

A semi-structured interview guide was used to moderate the focus group discussions (Supplementary file 2). To inform the development of the interview guide, we conducted a systematic literature search to identify similar studies [17] and found one previous focus group study that focused solely on drug administration [24]. Based on the literature review [17], the basic principles of applying focus group methodologies in pharmacy practice [30], and the research team's expertise, a semi-structured interview guide was developed to align with the research objective. It comprised six open-ended questions focused on user experience, perceptions of barcode technology, facilitators, barriers, medication safety considerations, and ideas for improvement (Supplementary file 2). The interview guide was deemed suitable in a pilot interview and used unaltered in the subsequent focus groups. The pilot interview was also included in the study materials. The interviews were audiotaped using Microsoft Teams. One member of the research team (LL) transcribed the audio files using the Microsoft Teams transcription tool and carefully reviewed and corrected all inaccuracies manually. After three discussions, the saturation point was reached because the same central issues kept recurring.

### Analysis

**Fig. 2:**
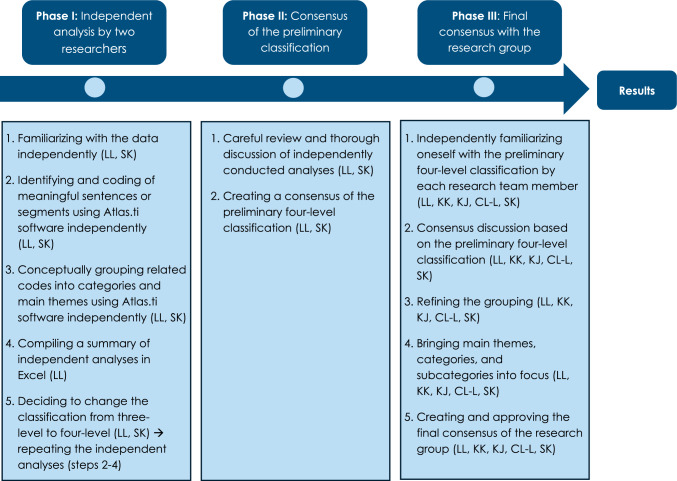
An overview of the inductive qualitative content analysis of the focus group discussions

The study material was analyzed using inductive qualitative content analysis (Fig. 2) [32]. We chose a data-driven approach because it is recommended when little qualitative information is available about the phenomenon under study and the field is fragmented [32]. The analysis was conducted using Atlas.ti and Excel software (Fig. 2). In the first phase, two researchers (LL, SK) independently analyzed the study material. In the second phase, the two researchers agreed on the preliminary classifications. Finally, the entire research group (LL, KK, CL-L, KJ, SK) reached consensus on the qualitative content analysis results.

### Ethics approval

Approval for the study was obtained from HUS. According to the Finnish National Board on Research Integrity TENK, ethical approval is not required for the present qualitative focus group study involving healthcare professionals [33]. This study was not a medical study that interfered with a patient’s physical or mental integrity, as defined by the Finnish Act on Medical Study (1999/488). The participants received a written statement about the study and provided written informed consent to participate before the focus group discussions. The research material was anonymized during data transcription and handled confidentially, so that only members of the research group who had signed the confidentiality and data protection agreement had access to it.

## Results

A total of 14 female participants from a wide age range were recruited into three focus groups. (Table 1). Each group consisted of 4 to 6 pediatric clinical pharmacists, and the focus group discussions lasted approximately 60 min (50–65 min). Most study participants had used barcode technology for over 2 years, although a few were new users.

**Table 1:** Characteristics of the study participants

Variable	Pediatric clinical pharmacists (*n*)
*Gender*	
Female	14
Male	0
*Age (years)*	
20–29	7
30–39	3
40–49	4
*Experience in the use of barcode technology (months)*	
0–6	1
7–12	2
13–18	1
19–24	2
Over 24	8

### Main themes arising from the study sample

**Fig. 3:**
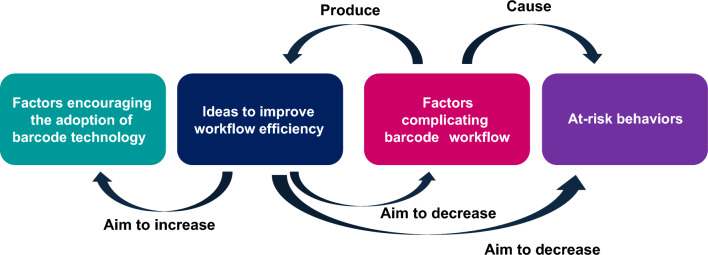
The main themes identified from the study material and their interconnections

Four main themes emerged from the data-driven analysis (Fig. 3). These themes were: factors encouraging the adoption of barcode technology, factors complicating barcode workflow, ideas to improve workflow efficiency, and at-risk behaviors. The study revealed that these main themes were closely interconnected (Fig. 3).

### Factors encouraging the adoption of barcode technology

**Fig. 4:**
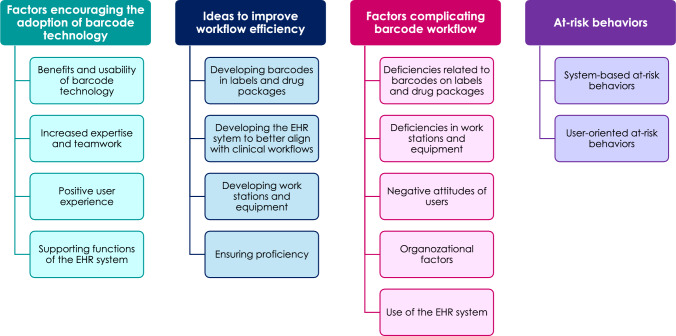
The main themes and their representative subcategories were derived from the study material. EHR = electronic health record

The first main theme, factors encouraging the adoption of barcode technology, was further divided into four subcategories (Fig. 4, Table 2). The *benefits and usability of barcode technology* included the development of EHR system functionalities, improved order templates, and an increased number of functioning barcodes. Using barcodes was perceived to save time, for example, by replacing a manual double-check performed by another colleague to verify the correct medication. The good usability of barcode scanners was noted to facilitate barcoding workflows, such as using the scanner in automatic reading mode, ensuring scanner availability, and easily moving them between workstations (for example, to replace an inoperative device). Increased patient and medication safety was also identified as one of the most significant advantages, as pharmacists felt that barcode workflows enhanced medication safety, promoted safe working practices, and increased confidence in ensuring the right patient, medication, dosage, route, and time.

**Table 2:** Examples of relevant categories, subcategories, significant sentences or segments, and citations within the four main themes identified in the study material

An example of a citation	Coding of meaningful sentences or segments	Subcategory	Category
*Factors encouraging the adoption of barcode technology*			
"[Barcode scanning] ensures that the package or product being processed matches the order and the medication intended for the patient.” – Group discussion 3	Barcode scanning ensures accuracy regarding the correct product (e.g., medication, diluent)	Barcoding guarantees that the correct medication is administered to the right patient	Benefits and usability of barcode technology
"When we want to prepare a medicine in a specific way and follow a particular order template, it may have given doctors a better understanding for prescribing medications.” – Group discussion 3	Enhanced understanding among doctors regarding the appropriate content of a medication order	Orientation and understanding of the EHR system and barcoding workflows	Increased expertise and teamwork
"– when you're completely new here [at the job] and there are many new medications, it provides you with the confidence that once you've scanned the preparation, at least you know it's the right one.” – Group discussion 3	The use of barcoding instills a sense of security for students and new employees (e.g., many new products, ensuring that the medications are correct)	User's sense of security	Positive user experience
"I truly appreciate the idea that the information will stay in the EHR system. You can then see afterwards, what has been done." – Group discussion 1	Information regarding medication preparation can be obtained from the EHR system afterwards (e.g., the components used, the individual performing the preparation)	Drug preparation is recorded in the EHR system	Supporting functions of the EHR system
*Factors complicating barcode workflow*			
"If there is medication that needs to be stored in the refrigerator, it becomes challenging when the ampoule lacks a barcode. In that case, you hesitate to take the entire package [out of the refrigerator], because then the rest will be left at room temperature." – Group discussion 2	The primary packaging of the medication (e.g., ampoule, vial) lacks a barcode, and the cardboard box must be stored in the refrigerator to prevent the medicine from warming and expiring	The primary packaging of the medication lacks a barcode	Deficiencies related to barcodes on labels and drug packages
"— we had a broken printer, so it printed a few lines [to the label] in a compressed manner, which caused the QR code to be partially wrinkled, so it didn't work in the medicine room [during preparation] and definitely wouldn't have worked in administration at the bedside either.” – Group discussion 3	The label is printed incorrectly (for example, the barcode is not positioned correctly or is printed only partially)	Challenges related to the use and functionality of label printers	Deficiencies in workstations and equipment
"Sometimes the EHR system displays such incorrect information that it suggests a preparation method it shouldn't. This can somehow demoralize the nurses. If that's the case, then why should we scan anything at all?” – Group discussion 2	Mistaken instructions in the EHR system lead to stress and distrust of the system	Negative user attitudes and perceptions of barcode workflows	Negative attitudes of users
"– for some nurses, it may be that the orientation is non-existent and then it is just assumed that you can automatically use the mobile device without any assistance." – Group discussion 3	Inadequate training on using new equipment (e.g., barcode scanner, mobile device, label printer)	Inadequate training and guidance	Organizational factors
"I really dislike how many different windows open, along with all these work steps and the constant clicking. It just feels overwhelming." – Group discussion 1	The barcoding workflow involves excessive clicking, pop-up windows, and numerous work steps	The laboriousness and complexity of the barcoding workflow	Use of the EHR system
*Ideas to improve workflow efficiency*			
"Well, first of all, for those [medications] without any barcode, it would be nice to have that code — and preferably, [the barcode] should be usable in the barcoding workflow." – Group discussion 3	Incorporating functional barcodes on all products and primary packaging (e.g., ampoules)	Creating medication packages and labels appropriate for barcode workflows	Developing barcodes in labels and drug packages
– it would be nice if the system could also evaluate the expiration date information somehow—right now, it does not notify if the ampoule is somehow expired." – Group discussion 2	The EHR system would notify users of an expired product during barcode scanning	Development of the structure and functions of the EHR system to meet clinical needs	Developing the EHR system to better align with clinical workflows
"—you should select the appropriate type of barcode scanner to meet the needs of a specific workstation, whether it has a fully connected electrical cord or is powered by a battery –" – Group discussion 1	A barcode scanner that is suitable for the workstation (e.g., wireless or station-mounted)	Improving workstations to be more suitable for barcode workflows	Developing workstations and equipment
"Perhaps during the orientation phase, in a way, instead of emphasizing that scanning the barcodes yields accurate results and good statistics, you should focus on the reasons behind it.” – Group discussion 3	Highlighting the medication safety benefits of barcode workflows as part of orientation	Development of orientation and guidelines	Ensuring proficiency
*At-risk behaviors*			
"If the primary packaging, such as an ampoule or a bottle, lacks a barcode, the barcodes are collected from those cardboard packages. [The barcodes cut from packages] are stored in the medicine room, and then we simply just use them.” – Group discussion 3	Barcodes cuts from the cardboard packaging of medications are kept in the medication room so they can be scanned during drug preparation	Problems with barcodes and equipment are hindering the use of workflows	System-based at-risk behaviors
"—First, we print the preparation and repackaging label, scan the components [used in preparation] in one place, and then move to another room to actually carry out the preparation. And then [during preparation] we rely on memory to recall what needs to be added and how much, meaning that scanning does not provide much safety when it isn't done simultaneously with preparation." – Group discussion 1	The barcodes of the components are scanned to the EHR system before conducting the preparation, after which the drug is prepared without utilizing the instructions provided by the EHR system	Completing the steps of barcoding workflows in the wrong order, at the wrong time, or incompletely	User-oriented at-risk behaviors

The study participants felt that using barcoding contributed to *increased expertise and teamwork* (Fig. 4, Table 2). Implementing barcode technology promoted multidisciplinary cooperation, for example, between clinical pharmacists and doctors, by making it easier for pharmacists to intervene in prescribing errors. Discussions highlighted improvements in doctors' ability to create more accurate and complete medication orders, a requirement for using barcode technology. Therefore, nurses or pharmacists were no longer responsible for choosing the drug formulation or administration method.

The *positive user experience* was highlighted as a facilitator (Fig. 4, Table 2). Participants reported that improved security measures to ensure the correct medication were beneficial to both students and new employees, as well as to more experienced clinical pharmacists. Although barcode workflows were found to take more time than earlier methods, users perceived their benefits as more significant. Many participants described instances in which problems with barcode workflows created uncertainty among employees.

*"-- I believe it is quite unsafe to proceed with something if you can't scan [the barcodes] --"*

- Group Discussion 3

*"-- if it [barcoding] hasn't worked there, then they are likely struggling --"*

- Group Discussion 1

The *supporting functions of the EHR system* that promote safe medication preparation and administration were another key facilitator (Fig. 4, Table 2). A barcode-assisted intravenous (IV) preparation workflow within the EHR system was found useful because it enabled detailed documentation and guided users through the correct drug preparation process, such as visually indicating the proper medications during barcode scanning. The execution of error-prone manual tasks, such as double-checking procedures and verifying product shelf life, was more reliable when supported by technology. The integration between the EHR system and automated dispensing cabinets was considered functional, particularly for the barcode-assisted dispensing workflow for solid oral medications.

### Factors complicating barcode workflow

The second main theme, factors complicating barcode workflow, was divided into five subcategories (Fig. 4, Table 2). *Deficiencies related to barcodes on labels and drug packages* included the complete absence of a barcode on the medicine packaging, medications with barcodes only on the outer cardboard packaging, and instances where the barcode on a package or label was difficult to scan or had been destroyed.

For example, barcodes placed on a transparent glass background or on a curved surface were often challenging to read with a barcode scanner. Participants also reported confusion associated with products containing additional non-functional codes. If it was not known which barcodes worked, users did not have time to try each one and might end up bypassing the barcode workflow.

*Deficiencies in workstations and equipment* were associated with poorly designed workstations, including inadequate tools, limited working space, and essential equipment, such as label printers and barcode scanners, placed too far apart (Fig. 4, Table 2). Additionally, issues concerning barcode scanners, such as poor scanning capability and insufficient battery life for extended use in a biological safety cabinet, were noted. On the other hand, label printers were seen as complex devices that sometimes produced incorrect labels (e.g., printing an unreadable barcode due to a wrinkled label).

*Negative attitudes of users* towards new practice guidelines and changes in clinical workflows were observed (Fig. 4, Table 2). The group discussions highlighted variations in approaches and attitudes toward barcode technology across professional groups. The prolonged problem-solving was found frustrating, such as when a non-functioning barcode had to be updated to the EHR system. The study material revealed instances in which barcode workflows could have been implemented but were neglected. For example, the barcode workflow was occasionally bypassed when administering medication to a sleeping patient because of concerns that the patient might wake up from the scanner's sound or light.

*Organizational factors* included inadequate training, competence, and management (Fig. 4, Table 2). Participants emphasized the user’s limited proficiency with the EHR system and with equipment such as barcode scanners and label printers. They believed the training and instructions for barcode workflows were inadequate, with some reporting that no training had been provided. The differing practices across care units within the department were also perceived as problematic.

The *use of the EHR system* was associated with challenges due to poor design and integration with other technological solutions (Fig. 4, Table 2). These issues included a complex, slow login process and the need to use a social and healthcare professional card to complete the preparation and repackaging functions required for pediatric IV stock solutions. Some shortcomings of the EHR system were even found to contribute to MEs, including challenges in prescribing. These included selecting an adult medication order template for a young patient and the inability of barcode workflows to ensure the correct amount of medicine was measured for the patient-specific dose (e.g., splitting a tablet in half). Challenges also arose when using barcode workflows in emergencies and other exceptional situations, such as borrowing medications from another care unit, where pharmacists often bypassed the workflow because it was slow and laborious.

### Ideas to improve workflow efficiency

The third main theme, ideas to improve workflow efficiency, was further divided into four subcategories (Fig. 4, Table 2). *Developing barcodes on labels and drug packages*, including a functional barcode on all commercial primary packages such as ampoules and injection vials, was a key area of development. Additionally, it was recommended that the batch number and expiration date should be included in all barcodes so that the EHR system could automatically verify and record this information during barcode scanning.

The participants emphasized *developing the EHR system to better align with clinical workflows* (Fig. 4, Table 2). This involved creating a more suitable EHR system structure, such as streamlining the login process and enabling barcode workflows across all medication administration scenarios, including emergencies and borrowing medication from other care units that do not use barcode technology. It was suggested to enhance the secondary use of information recorded during barcoding workflows, such as retrospectively identifying patients who received a batch linked to a product defect.

The study material highlighted the need for *developing workstations and equipment* (Fig. 4, Table 2)*.* The proposals included development ideas for devices introduced in clinical areas during the implementation period, such as barcode scanners, label printers, and mobile devices. For example, there were expectations for longer battery life in scanners and upgraded devices to improve performance inside a biological safety cabinet. The use of new technologies, such as an eye-tracking sensor for preparing medication instead of a computer mouse, was suggested. It was deemed essential to provide sufficient equipment and workspace, including installing appropriate equipment in isolation rooms to facilitate barcode workflows. Furthermore, it was proposed that workstation designs should include verifying the appropriateness of the equipment for the new workspace.

The ideas to improve workflow efficiency included suggestions for *ensuring proficiency* (Fig. 4, Table 2). Pharmacists emphasized the need for more precise technical instructions on using and maintaining devices, such as barcode scanners. Additionally, all discussions emphasized the importance of a comprehensive, adequately resourced orientation to the use of equipment and the EHR system.

### At-risk behaviors

The fourth main theme, at-risk behaviors, was divided into two subcategories (Fig. 4, Table 2). *System-based at-risk behaviors* included issues with the structure and functionality of the EHR system, such as the inability to multiply the composition of a patient-specific IV fluid to prepare a larger batch at once, resulting in the need for manual calculations. Furthermore, due to missing barcodes or malfunctioning equipment, the proper workflow had to be bypassed and replaced with workarounds, such as manually entering the product number or scanning a barcode cut from cardboard packaging stored in the medicine room.

*User-oriented at-risk behaviors* were connected to outdated workflows (Fig. 4, Table 2). These included replacing barcoding workflows with manual double-checking or with retrospective recording of medication administration at the nurse's station rather than at the bedside. Other examples included performing workflow steps in the wrong order (e.g., scanning components after the medication had been prepared) and incomplete workflow execution (e.g., scanning only one ampoule even though several had been used).

## Discussion

The focus groups involving clinical pharmacists revealed various complex facilitators and barriers to barcode technology across multiple hospital functions. As observed elsewhere, pharmacists also highlighted improved medication safety, reliability, and effectiveness as key facilitators, making them unwilling to revert to previous practices [24, 26, 28, 34–36]. While multidisciplinary teamwork has been acknowledged as crucial for implementation [24, 25, 27], our study further emphasized the role of pharmacists in problem-solving, developing workflow design, and establishing a solid foundation for barcode workflow by ensuring order accuracy. We identified several barriers to barcode technology, often leading to workarounds and poor compliance in clinical practice, a concern highlighted in other studies [17, 23–28, 34–39]. Our results also demonstrated the potential for user engagement in barcode workflow development, a key facilitator identified in other studies, as the focus groups generated several innovative ideas to overcome barriers and improve workflow efficiency [17, 23].

Compared with earlier studies that focused mainly on nurses’ perspectives [17, 22, 27, 28], our results highlighted observations on barcode-assisted medication dispensing and preparation workflows. Several systemic flaws associated with barcode-assisted aseptic IV preparation (e.g., illegible or absent barcodes on primary packaging, equipment unsuitable for use in biological safety cabinets) were identified as critical barriers requiring workarounds to complete drug preparation. Similar issues with devices and network connectivity have been observed in drug administration, underscoring the need for carefully designed workstations and selecting the most suitable equipment for different tasks within the barcode-assisted medication management process [17, 22, 24, 26–28, 34, 36, 39]. The deficiencies in barcodes on labels and drug packages were identified as a key challenge and area for improvement, likely because of our hospital’s pioneering role in Finland in introducing barcode technology. Addressing this issue to support the adoption of barcode workflows in clinical practice requires efforts at both the national and hospital levels [17]. These efforts include mandating barcode labels for all commercial dosage forms and containers, establishing an organizational escalation process for reporting and resolving barcode scanning failures, and implementing unit-dose dispensing systems [17, 40–42].

The pharmacists identified pediatric-specific barriers resulting from individualized dosing and complex calculations, which have been reported to complicate the entire medication management process in pediatric hospitals [43, 44]. Challenges in prescribing, such as using an adult order template for a young patient, were associated with a higher risk of MEs by impairing the usability of barcode workflows. Furthermore, pediatric dosing required additional steps, including completing two separate preparation processes for the stock solution and the patient-specific dose, and performing manual calculations to prepare a larger batch of patient-specific IV fluid or parenteral nutrition. The pharmacists noted the limitations of barcode technology in ensuring accurate measurements for small, customized doses, such as drawing medicine into syringes or cutting tablets. Because many pediatric MEs are associated with incorrect doses [43, 45, 46], implementing additional safety measures alongside barcode technology to ensure correct dosing is necessary. In a children's hospital, maintaining a pediatric drug formulary and age-group-specific order templates through a systematic pharmacy informatics process is essential to facilitate the effectiveness of barcode technology [1, 20].

The focus groups emphasized that properly implementing barcode workflows requires users to have a high level of expertise in system functionality and workflows. Although barcode technology had been in use for two years, clinical pharmacists still reported negative attitudes toward it as a significant barrier. In other studies, resistance to change or even fear of new practices has been reported [24, 26, 27]. Additionally, inadequate training, management, and response to observed challenges in clinical work were found problematic, as also reported elsewhere [17, 24, 25, 27, 34, 39]. Deficiencies in the EHR system structure and medication order templates were identified as contributing factors to MEs, which tend to recur until the issue is corrected. These findings highlight the challenges of implementing the highest-leverage systemic defenses in clinical practice, emphasizing the importance of ongoing risk management processes for EHR system maintenance and development [5, 47–49]. Because barcode workflows generate more accurate documentation through the medication management process, the secondary use of this data offers an opportunity to support daily operations, research, and reporting [47, 50, 51]. However, addressing the barriers and development ideas identified in the focus groups is critical to ensure high-quality documentation.

Our study has several limitations. Although we used the COREQ checklist to ensure proper reporting and to consider factors that might affect reliability, the results of a qualitative focus group study cannot be generalized statistically without additional quantitative research [29, 30]. However, selecting a qualitative method was justified because our study aimed to develop a preliminary understanding of a little-studied phenomenon [30, 31]. Because the number of participants tends to be small, the quality of the study material depends heavily on the careful planning of the focus group discussions [29, 30]. Therefore, we conducted a systematic literature search to summarize earlier qualitative studies to inform the planning process [17], aimed to interview all pediatric pharmacists who met the participation criteria, and conducted a pilot study to validate the semi-structured interview guide. However, because the study participants were clinical pharmacists experienced with barcode workflows, investigating the perspectives of other healthcare professionals and pharmacists with limited practical experience with this technology remains an important area for future research. Inductive qualitative content analysis lacks an established theoretical framework and relies on subjective interpretation by researchers, which should be considered when interpreting our results [29, 32]. To address these limitations, two researchers with different backgrounds (LL, SK) conducted the qualitative content analysis independently, followed by a thorough review of the results with the entire research team. (LL, KK, KJ, CL-L, SK). Although based on Finland’s largest children’s hospital, providing highly specialized care, our study is influenced by the specific patient population, local operational models, pharmaceutical care structures, and the division of tasks among different professional groups.

Our results can be used to improve the usability of barcode workflows in pediatric hospitals. Some of our findings, such as observations on workstation design, barcode availability, and organizational factors, may also apply to adult hospitals. Although barcode technology is widely recommended as a high-level systemic defense, successful implementation in clinical practice requires organizational commitment to ongoing risk management and EHR system maintenance [6–8, 17, 23]. Our study was unable to consider the perspectives of patients, caregivers, or other professional groups, which are important areas for future research. Additionally, scientific evidence on interventions that promote user compliance and on effective organizational models to improve the usability of barcode technology is needed.

## Conclusion

Our study summarizes clinical pharmacists' perceptions of the facilitators and barriers to using barcode technology, an area of research that is relatively new. Although the focus groups highlighted improved medication safety as a key facilitator, they also identified several barriers that require further development. Establishing an organizational process to address system deficiencies and enhance the usability of barcode technology is essential to prevent workarounds that compromise medication safety. In addition to prior studies, our research highlighted issues related to dispensing and preparation workflows and the critical need for pediatric-specific customization of EHR systems. Our findings can inform risk management in the implementation and maintenance of barcode technology.

## Supplementary Information

Below is the link to the electronic supplementary material.

Supplementary file1 (DOCX 22 kb)

Supplementary file2 (DOCX 16 kb)

## Data Availability

The datasets generated and analyzed during the current study are not available due to the confidential nature of the focus group discussions, as the participants were assured that raw data would not be shared.
