# Supplementary material for: Facilitators and barriers in using barcode technology to ensure safe medication dispensing, preparation, and administration in a children's hospital: a focus group study for clinical pharmacists
Source: Int J Clin Pharm. 2026 Apr 2;48(4):1519–32. doi: 10.1007/s11096-026-02132-0 (PMC13369583; doi:10.1007/s11096-026-02132-0)
Supplement: Supplementary file 1 — Supplementary file1 (DOCX 22 kb) [file 11096_2026_2132_MOESM1_ESM.docx]

**Title:** Facilitators and barriers in using barcode technology to ensure safe medication dispensing, preparation, and administration in a children's hospital – A focus group study for ward pharmacists

**Authors:** Laura Laakkonen ^a^, Kirsi Kvarnström ^ab^, Katja Janhunen ^c^, Carita Linden-Lahti ^ab^, Sini Kuitunen ^ab^

**Affiliations:** ^a^ Division of Pharmacology and Pharmacotherapy, Faculty of Pharmacy, University of Helsinki, Finland.

^b^ HUS Pharmacy, Helsinki University Hospital (HUS), Helsinki, Finland.

^c^ Corporate Group Administration, Western Uusimaa Wellbeing Services County, Finland.

**Correspondence:** Sini Kuitunen, Division of Pharmacology and Pharmacotherapy, Faculty of Pharmacy, University of Helsinki, Viikinkaari 5 E, PL 56, 00014 Helsingin yliopisto, Finland. Email address: sini.kuitunen@helsinki.fi.

**Journal:** International Journal of Clinical Pharmacy

**Supplementary material:** Consolidated criteria for reporting qualitative research (COREQ) tool.

| **Topic** | **Item No.** | **Guide Questions/ Description** | **Reported on Section (Manuscript page)** |
| --- | --- | --- | --- |
| **Domain 1: Research team and reflexivity** | | | |
| *Personal characteristics* | | | |
| Interviewer/facilitator | 1 | Which author/s conducted the interview or focus group? | Data collection and preprocessing (5–6) |
| Credentials | 2 | What were the researcher’s credentials? E.g. PhD, MD | Data collection and preprocessing (5–6) |
| Occupation | 3 | What was their occupation at the time of the study? | Data collection and preprocessing (5–6) |
| Gender | 4 | Was the researcher male or female? | Data collection and preprocessing (5–6) |
| Experience and training | 5 | What experience or training did the researcher have? | Data collection and preprocessing (5–6) |
| *Relationship with participants* | | | |
| Relationship established | 6 | Was a relationship established prior to study commencement? | Data collection and preprocessing (5–6) |
| Participant knowledge of the interviewer | 7 | What did the participants know about the researcher? e.g. personal goals, reasons for doing the research | Data collection and preprocessing (5–6) |
| Interviewer characteristics | 8 | What characteristics were reported about the inter viewer/facilitator? e.g. Bias, assumptions, reasons and interests in the research topic | Data collection and preprocessing (5–6) |
| **Domain 2: Study design** | | | |
| *Theoretical framework* | | | |
| Methodological orientation  and Theory | 9 | What methodological orientation was stated to underpin the study? e.g. grounded theory, discourse analysis, ethnography, phenomenology, content analysis | Study design (4), Analysis (6), Figure 2 |
| *Participant selection* | | | |
| Sampling | 10 | How were participants selected? e.g. purposive, convenience, consecutive, snowball | Study participants (5) |
| Method of approach | 11 | How were participants approached? e.g. face-to-face, telephone, mail, email | Study participants (5) |
| Sample size | 12 | How many participants were in the study? | Study participants (5), Results (7), Table 1 |
| Non-participation | 13 | How many people refused to participate or dropped out? Reasons? | Study participants (5) |
| *Setting* | | | |
| Setting of data collection | 14 | Where was the data collected? e.g. home, clinic, workplace | Study setting (4) |
| Presence of nonparticipants | 15 | Was anyone else present besides the participants and researchers? | Data collection and preprocessing (5–6) |
| Description of sample | 16 | What are the important characteristics of the sample? e.g. demographic data, date | Results (7), Table 1 |
| *Data collection* | | | |
| Interview guide | 17 | Were questions, prompts, guides provided by the authors? Was it pilot tested? | Data collection and preprocessing (5–6), Supplementary file 2 |
| Repeat interviews | 18 | Were repeat inter views carried out? If yes, how many? | Data collection and preprocessing (5–6) |
| Audio/visual recording | 19 | Did the research use audio or visual recording to collect the data? | Data collection and preprocessing (5–6) |
| Field notes | 20 | Were field notes made during and/or after the interview or focus group | Data collection and preprocessing (5–6) |
| Duration | 21 | What was the duration of the interviews or focus group? | Results (7) |
| Data saturation | 22 | Was data saturation discussed? | Data collection and preprocessing (5–6) |
| Transcripts returned | 23 | Were transcripts returned to participants for comment and/or correction? | N/A |
| **Domain 3: analysis and findings** | | | |
| *Data analysis* | | | |
| Number of data coders | 24 | How many data coders coded the data? | Analysis (6), Figure 2 |
| Description of the coding tree | 25 | Did authors provide a description of the coding tree? | Factors encouraging the adoption of barcode technology (8–13), Factors complicating barcode workflow (13-15), Ideas to improve workflow efficiency (14), At-risk behaviors 15-16), Figure 4, Table 2 |
| Derivation of themes | 26 | Were themes identified in advance or derived from the data? | Analysis (6), Figure 3, Main themes arising from the study sample (8) |
| Software | 27 | What software, if applicable, was used to manage the data? | Analysis (6), Figure 2 |
| Participant checking | 28 | Did participants provide feedback on the findings? | N/A |
| *Reporting* | | | |
| Quotations presented | 29 | Were participant quotations presented to illustrate the themes/findings? Was each quotation identified? e.g. participant number | Factors encouraging the adoption of barcode technology (8–13), Table 2 |
| Data and findings consistent | 30 | Was there consistency between the data presented and the findings? | Factors encouraging the adoption of barcode technology (8–13), Factors complicating barcode workflow (13-15), Ideas to improve workflow efficiency (14), At-risk behaviors 15-16), Figure 4, Table 2 |
| Clarity of major themes | 31 | Were major themes clearly presented in the findings? | Factors encouraging the adoption of barcode technology (8–13), Factors complicating barcode workflow (13-15), Ideas to improve workflow efficiency (14), At-risk behaviors 15-16), Figure 3, Figure 4, Table 2 |
| Clarity of minor themes | 32 | Is there a description of diverse cases or discussion of minor themes? | Factors encouraging the adoption of barcode technology (8–13), Factors complicating barcode workflow (13-15), Ideas to improve workflow efficiency (14), At-risk behaviors 15-16), Figure 4, Table 2 |

**Reference:** Tong A, Sainsbury P, Craig J. Consolidated criteria for reporting qualitative research (COREQ): A 32-item checklist for interviews and focus groups. *Int J Qual Health Care*. 2007;19(6):349-357. doi:10.1093/intqhc/mzm042
