# Supplementary material for: Facilitators and barriers in using barcode technology to ensure safe medication dispensing, preparation, and administration in a children's hospital: a focus group study for clinical pharmacists
Source: Int J Clin Pharm. 2026 Apr 2;48(4):1519–32. doi: 10.1007/s11096-026-02132-0 (PMC13369583; doi:10.1007/s11096-026-02132-0)
Supplement: Supplementary file 2 — Supplementary file2 (DOCX 16 kb) [file 11096_2026_2132_MOESM2_ESM.docx]

**Title:** Facilitators and barriers in using barcode technology to ensure safe medication dispensing, preparation, and administration in a children's hospital – A focus group study for ward pharmacists

**Authors:** Laura Laakkonen ^a^, Kirsi Kvarnström ^ab^, Katja Janhunen ^c^, Carita Linden-Lahti ^ab^, Sini Kuitunen ^ab^

**Affiliations:** ^a^ Division of Pharmacology and Pharmacotherapy, Faculty of Pharmacy, University of Helsinki, Finland.

^b^ HUS Pharmacy, Helsinki University Hospital (HUS), Helsinki, Finland.

^c^ Corporate Group Administration, Western Uusimaa Wellbeing Services County, Finland.

**Correspondence:** Sini Kuitunen, Division of Pharmacology and Pharmacotherapy, Faculty of Pharmacy, University of Helsinki, Viikinkaari 5 E, PL 56, 00014 Helsingin yliopisto, Finland. Email address: sini.kuitunen@helsinki.fi.

**Journal:** International Journal of Clinical Pharmacy

**Supplementary material:** The semi-structured interview guide for the focus group discussions.

| **The purpose of these open-ended questions was to encourage discussion** |
| --- |
| - How long have you been using barcode technology in your daily work? - What are your thoughts on the medication workflows that primarily utilize barcodes? - What factors create challenges in using barcode technology? - What do you think are the advantages of using barcode technology? - How has barcode technology impacted patient safety? - In your opinion, how can the workflows of barcode-assisted medication administration and preparation be improved? |
